# Six sequence variants on chromosome 9p21.3 are associated with a positive family history of myocardial infarction: a multicenter registry

**DOI:** 10.1186/1471-2261-11-9

**Published:** 2011-03-07

**Authors:** Thomas Scheffold, Silke Kullmann, Andreas Huge, Priska Binner, Hermann R Ochs, Wolfgang Schöls, Joachim Thale, Wolfgang Motz, Franz Josef Hegge, Christoph Stellbrink, Thomas Dorsel, Hartmut Gülker, Hubertus Heuer, Wilfried Dinh, Monika Stoll, Georg Haltern

**Affiliations:** 1 Institute for Heart and Circulation Research, University of Witten/Herdecke, 44227 Dortmund, Germany; 2 Leibniz-Institute for Arteriosclerosis Research, University of Münster, 48149 Münster, Germany; 3 Department of Internal Medicine, Marienkrankenhaus Soest, 59494 Soest, Germany; 4 Department of Cardiology, Heart Centre Duisburg, 47137 Duisburg, Germany; 5 Department of Angiology, Heart Centre Duisburg, 47137 Duisburg, Germany; 6 Department of Cardiology, Schüchtermann-Klinik, Heart Centre Osnabrück-Bad Rothenfelde, 49214 Bad Rothenfelde, Germany; 7 Heart and Diabetes Centre Mecklenburg-Vorpommern, Klinikum Karlsburg, 17495 Karlsburg, Germany; 8 Department of Internal Medicine, St. Christophorus-Krankenhaus Werne, 59368 Werne, Germany; 9 Hospital for Cardiology and Internal Intensive Care, Städtische Kliniken Bielefeld-Mitte, 33604 Bielefeld, Germany; 10 Department of Cardiology, Josephs-Hospital Warendorf, 48231 Warendorf, Germany; 11 Heart Centre Wuppertal Helios-Kliniken, 42117 Wuppertal, Germany; 12 Department of Internal Medicine, St. Johannes Hospital Dortmund, 44227 Dortmund, Germany

## Abstract

**Background:**

Recent genome-wide association studies have identified several genetic loci linked to coronary artery disease (CAD) and myocardial infarction (MI). The 9p21.3 locus was verified by numerous replication studies to be the first common locus for CAD and MI. In the present study, we investigated whether six single nucleotide polymorphisms (SNP) rs1333049, rs1333040, rs10757274, rs2383206, rs10757278, and rs2383207 representing the 9p21.3 locus were associated with the incidence of an acute MI in patients with the main focus on the familial aggregation of the disease.

**Methods:**

The overall cohort consisted of 976 unrelated male patients presenting with an acute coronary syndrome (ACS) with ST-elevated (STEMI) as well as non-ST-elevated myocardial infarction (NSTEMI). Genotyping data of the investigated SNPs were generated and statistically analyzed in comparison to previously published findings of matchable control cohorts.

**Results:**

Statistical evaluation confirmed a highly significant association of all analyzed SNP's with the occurrence of MI (p < 0.0001; OR: 1.621-2.039). When only MI patients with a positive family disposition were comprised in the analysis a much stronger association of the accordant risk alleles with incident disease was found with odds ratios up to 2.769.

**Conclusions:**

The findings in the present study confirmed a strong association of the 9p21.3 locus with MI particularly in patients with a positive family history thereby, emphasizing the pathogenic relevance of this locus as a common genetic cardiovascular risk factor.

## Background

In recent years, genome-wide association studies (GWAS) have displayed an effective approach to localize genomic regions predisposing to common, polygenetic disorders, including cardiovascular disorders[1,2]. By means of independent genome-wide association studies using single nucleotide polymorphism (SNP) arrays the first common chromosomal locus has been identified which confers susceptibility for coronary artery disease (CAD) and myocardial infarction (MI)[3-5]. Initially, the two SNPs rs10757274 and rs2383206 consistently associated with CAD were identified in a large scale study population of Caucasian origin[3]. Both polymorphisms are located within a locus spanning a 58-kilobase region on chromosome 9p21.3. Three additional SNPs, rs1333040, rs2383207, and rs10757278 on the 9p21.3 locus and in genetic disequilibrium were identified independently as being associated with MI[4]. Moreover, large-scale genome-wide association scans identified further polymorphisms, amongst others rs1333049, within the same genomic region to be associated with MI and CAD and therefore verified chromosome 9p21.3 as a susceptibility locus for the incidence of the disease[5,6]. Based on these findings, replication studies using case-control designs proved that the identified SNPs were associated with CAD and MI in a large variety of study population[7-17]. In all studies, the association of the 9p21.3 locus with CAD and MI was shown to be independent of conventional risk factors. In a subsequent study the association of the rs1333049 risk variant with the extend of CAD could be established[18]. Thus, even though the responsible gene in this region is still unknown, the 9p21.3 locus was verified to be the first common genetic factor affecting the risk of CAD and MI. Further studies may provide new insights into the impact of chromosome 9p21.3 on the pathogenesis of CAD and the occurrence of a MI.

In the present study the influence of six SNPs (rs1333049, rs1333040, rs10757274, rs2383206, rs10757278, and rs2383207) representing the 9p21.3 region on the occurrence of MI was investigated. The main focus of our investigation was on an association of the 9p21.3 locus with a positive family history of MI in a large cohort of patients presenting with an acute coronary syndrome (ACS).

## Methods

### Study subjects

Over a three year span (2004 to 2006), a total of 976 unrelated male patients younger than 65 years were enrolled into a study due to a symptomatic ACS with STEMI as well as NSTEMI. This prospective multi-centre registry involved four heart centres and six cardiologic departments in Germany. The study was approved by the ethical committee of the University Witten/Herdecke, Germany, and conformed to the declaration of Helsinki. All participants gave written informed consent to participation.

MI was defined as follows: ischemic type chest pain lasting for more than 20 minutes, at least 0.1 mV of ST-segment elevation in the limb leads and/or at least 0.2 mV elevation in the precordial leads and one of the following criteria: left bundle branch block, new Q wave (at least 0.03 s), elevated creatine kinase or positive troponin T or I. All cases underwent cardiac catheterization and interventional or surgical revascularization. Patients < 18 and > 65 years, with unstable angina or without central European origin were excluded from further studies.

### Blood samples and DNA preparation

EDTA-blood samples were drawn from each participant at baseline ward round. Genomic DNA was extracted from 350 μl of these samples using the BioRobot EZ1 and the EZ1 blood extraction kit according to the manufacturer's instructions (QIAGEN; Hilden, Germany). DNA was quantified using the BioPhotometer (Eppendorf; Hamburg, Germany) and each sample was diluted to a final concentration of 25 ng/μl.

### PCR amplification and genotyping

Genotyping of the investigated SNPs (rs1333049, rs1333040, rs10757274, rs2383206, rs10757276, and rs2383207) was carried out by real time PCR and subsequent melting curve analysis on a LightCycler 480 instrument (Roche Applied Science; Mannheim, Germany). The primers and hybridization probes were designed and synthesized by Tib MolBiol GmbH (Berlin, Germany). PCR was carried out in 96-well plates (Roche Applied Science; Mannheim, Germany) using 12.5 ng of genomic DNA as template in a final reaction volume of 5 μl. Reaction mixtures contained 0.5 μM of each primer, 0.15 μM of SNP-specific hybridization probes and 1 μl of LightCycler 480 Genotyping Master (Taq DNA polymerase, reaction buffer, 15 mM MgCl_2_, and a dNTP mixture with UTP instead of dTTP) (Roche Diagnostics GmbH, Mannheim, Germany).

The cycling program consisted of 10 minutes of initial denaturation at 95°C, followed by 35 cycles (rs1333049, rs2383206, rs2383207, rs10757274, rs10757278) or 40 cycles (rs1333040) of denaturation at 95°C for 5 seconds, annealing at 53°C (rs1333049, rs2383206, rs2383207), at 59°C (rs1333040) or at 62°C (rs10757274, rs10757278) for 10 seconds, and extension at 72°C for 10 seconds. After PCR melting curves were generated by holding the reaction mixture at 95°C for 1 minute, stepwise lowering of the temperature to 65°C, 55°C and 45°C, holding for 30 seconds at each temperature, lowering to 40°C for 2 minutes, followed by continuously heating to 75°C. Melting curve analyses were conducted using the LightCycler 480 software according to the manufacturer's instructions (Roche Diagnostics GmbH; Mannheim, Germany).

### Statistical analysis

The distribution of genotypes and the allelic frequencies were investigated and analyzed by alignment to previously published data of applicable controls. Genotypes were tested for Hardy-Weinberg equilibrium among MI cases and controls using a chi-square test with one degree of freedom. Differences in the genotype distribution were tested for statistically significance and odds ratios (ORs) were determined using the 2-way contingency table chi-square test using a freely accessible program (http://statpages.org/ctab2x2.html). For all data, the association was considered to be significant for p < 0.05.

## Results

### Patients

A total of 976 patients, aged 24 to 65, were included into the present study to investigate the association between the 9p21.3 locus polymorphisms and acute MI. 72.8% of the ACS patients hospitalised with MI were diagnosed with ST-segment elevation (STEMI), 27.2% of the patients with non ST-segment elevation (NSTEMI). Of 976 patients 361 patients (37%) declared a positive family history of MI, defined as at least one first-degree relative affected before the age of 60 years, 404 patients (41.4%) had a negative family history, whereas 211 patients (21.6%) were not aware of another MI case in their family.

Controls were selected from previously published genotyping data. These data comprised subjects from Europe which were enrolled for independent studies as previously described elsewhere[3,4,11]. The baseline clinical characteristics and procedural parameters of the study population are presented in Table 1.

**Table 1:** Clinical characteristics of the study population

Characteristic	Study Population n = 976
**ACS -- no. (%)**	
STEMI	711 (72.8)
NSTEMI	266 (27.2)
**Age at recruitment -- yr**	52 ± 8,7
≤ 55	601 (61.6)
**Cardiovascular risk factor -- no. (%)**	
Diabetes mellitus	131 (13.4)
Hypertension	507 (51.9)
Hyperlipidaemia	529 (54.2)
Former or current smoker	843 (86.4)
BMI -- kg/m^2^	28 ± 4.97
BMI ≥ 30 kg/m^2^	280 (28.7)
**Positive family history of MI -- no. (%)**	361 (37.0)
**Event -- no. (%)**	
Previous MI	
> 14 days	99 (10.1)
< 14 days	9 (0.9)
CAD	156 (16.0)
PCI	111 (11.4)
CABG	27 (2.8)
Stroke	14 (1.4)
PAOD	26 (2.6)

Data are shown as mean ± standard deviation; *MI *myocardial infarction, *STEMI *ST-elevation myocardial infarction, *NSTEMI *non ST-elevation myocardial infarction, *CAD *coronary artery disease, *PCI *percutaneous coronary intervention, *BMI *body mass index, *CABG *coronary artery bypass grafting, *PAOD *peripheral artery occlusive disease.

### Replicated association of six variants on 9p21.3 with acute MI

Six SNPs on chromosome 9p21.3 were selected for genotyping based on strength of association in previous studies[3,4,11]. In order to confirm the reported association of the investigated SNPs with MI, genotyping data of the overall study cohort were compared to previously published data of control subjects (Table 2). All controls were without known history of CAD. Furthermore, only control cohorts were selected, which were comparable to the MI cases of the present study concerning geographic origin (northern and central Europe), age and wherever applicable, gender.

**Table 2:** Association of six variants on 9p21.3: MI study population vs. controls

Variant	Controls		Risk Allele Frequency	MI Cases n = 976		Risk Allele Frequency	HW	P value	OR (95% CI)
**rs1333040**	b) n = 3532								
	C/C	T/T	T: 0.50	C/C	T/T	T: 0.56	0,993	<0.0001	1.621 (1.317-2.994)
	893	889		181	292				
									
**rs10757274**	c) n = 9053								
	A/A	G/G	G: 0.45	A/A	G/G	G: 0.52	0,075	<0.0001	1.904 (1.570-2.309)
	2752	1758		208	253				
									
**rs2383206**	c) n = 9053								
	A/A	G/G	G: 0.47	A/A	G/G	G: 0.55	0,06	<0.0001	1.867 (1.535-2.272)
	2489	1981		183	272				
									
**rs2383207**	b) n = 3532								
	A/A	G/G	G: 0.46	A/A	G/G	G: 0.55	0,25	<0.0001	2.039 (1.654-2.514)
	1016	746		183	274				
									
**rs10757278**	b) n = 718								
	A/A	G/G	G: 0.43	A/A	G/G	G: 0.52	0,062	<0.0001	1.941 (1.458-2.583)
	224	128		211	243				
									
**rs1333049**	a) n = 999								
	G/G	C/C	C: 0.46	G/G	C/C	C: 0.52	0,233	<0.0001	1.653 (1.280-2.135)
	292	205		212	246				

Genotype distribution and allelic frequencies of the investigated SNPs on 9p21.3 of the overall study cohort were compared with control data of the PopGen cohort (a) [11], the Iceland B collective (b) [4] or the Copenhagen City Heart Study (c) [3]. HW: P value for Hardy-Weinberg equilibrium test. *P *values and Odds ratios (OR) were calculated for the high-risk homozygous alleles.

The rs1333049 genotype distribution and allelic frequencies were compared to the data of the PopGen controls[11]. For analysing the data of rs1333040, rs10757278, and rs2383207 we used the controls of the Iceland B collectives [4] and for the analysis of rs2383206 and rs10757274 the Copenhagen City Heart Study (CCHS) controls[3]. The genotype distribution in MI cases and controls did not significantly (P > 0.1) deviate from the Hardy-Weinberg equilibrium.

As shown in table 2, a statistically significant difference in the distribution of genotypes of MI cases compared to controls was observed for all analyzed SNPs (p < 0.0001). Odds ratios determined for the respective high-risk homozygous alleles were in the range of 1.621 to 2.039. These findings verified the strong association of the 9p21.3 locus with MI in this large scale study population of male patients presented with ACS.

### Variants on 9p21.3 are associated with a positive family history of MI

To investigate the association between the sequence variants and a positive family history of MI, patients with at least one first degree-relative who had suffered from a premature MI (n = 361) were selected and the genotype distribution and allelic frequencies for all variants were analyzed and compared with the data of the corresponding control cohorts (Table 3) and with the data of the patients without a positive family history (Table 4).

**Table 3:** Association of six variants on 9p21.3: MI study subpopulation with a positive family history vs. Controls

Variant	Controls		Risk Allele Frequency	Positive Family History		Risk Allele Frequency	P value	OR (95% CI)
**rs1333040**	b) n = 3532							
	C/C	T/T	T: 0.50	C/C	T/T	T: 0.57	<0.0001	1.892 (1.366-2.619)
	893	889		60	113			
								
**rs10757274**	c) n = 9053							
	A/A	G/G	G: 0.45	A/A	G/G	G: 0.56	<0.0001	2.625 (1.922-3.585)
	2752	1758		65	109			
								
**rs2383206**	c) n = 9053							
	A/A	G/G	G: 0.47	A/A	G/G	G: 0.57	<0.0001	2.351 (1.719-3.214)
	2489	1981		62	116			
								
**rs2383207**	b) n = 3532							
	A/A	G/G	G: 0.46	A/A	G/G	G: 0.58	<0.0001	2.570 (1.864-3.543)
	1016	746		62	117			
								
**rs10757278**	b) n = 718							
	A/A	G/G	G: 0.43	A/A	G/G	G: 0.55	<0.0001	2.769 (1.904-4.026)
	224	128		67	106			
								
**rs1333049**	a) n = 999							
	G/G	C/C	C: 0.46	G/G	C/C	C: 0.55	<0.0001	2.232 (1.567-3.180)
	292	205		67	105			

Genotype distribution and allelic frequencies of the investigated SNPs on 9p21.3 of the study subpopulation with a positive family history compared to control of the PopGen cohort (a) [11], the Iceland B collective (b) [4] or the Copenhagen City Heart Study (c) [3]. *P *values and Odds ratios (OR) were calculated for the high-risk homozygous alleles.

**Table 4:** Association of six variants on 9p21.3: MI study subpopulation with a positive family history vs. MI study subpopulation with a negative family history

Variant	Negative Family History n = 404		Risk Allele Frequency	Positive Family History n = 361		Risk Allele Frequency	P value	OR (95% CI)
**rs1333040**								
	C/C	T/T	T: 0.53	C/C	T/T	T: 0.57	0.102	1.426(0.932-2.180)
	81	107		60	113			
**rs10757274**								
	A/A	G/G	G: 0.48	A/A	G/G	G: 0.56	0.002	1.940(1.269-2.964)
	96	83		65	109			
**rs2383206**								
	A/A	G/G	G: 0.51	A/A	G/G	G: 0.57	0.015	1.703(1.108-2.618)
	81	89		62	116			
**rs2383207**								
	A/A	G/G	G: 0.51	A/A	G/G	G: 0.58	0.016	1.696(1.103-2.608)
	80	89		62	117			
**rs10757278**								
	A/A	G/G	G: 0.47	A/A	G/G	G: 0.55	0.003	1.923(1.256-2.944)
	96	79		67	106			
**rs1333049**								
	G/G	C/C	C: 0.48	G/G	C/C	C: 0.55	0.003	1.896(1.241-2.898)
	98	81		67	105			

Genotype distribution and allelic frequencies of the investigated SNPs on 9p21.3 of the study subpopulation with a positive family history were compared with the study subpopulation with a negative family history. *P *values and Odds ratios (OR) were calculated for the high-risk homozygous alleles.

**Figure 1:**
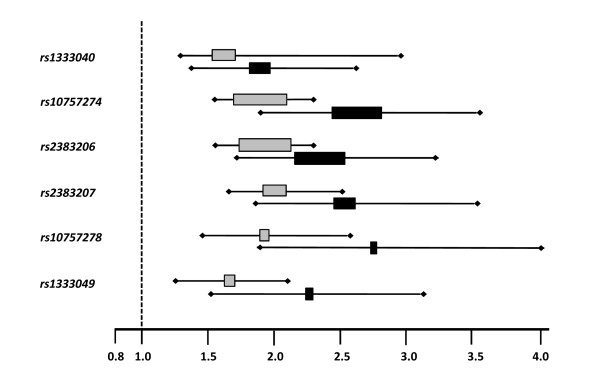
**Homozygous Odds Ratios and 95% Confidence Intervals for the investigated SNPs**. Boxes indicate the relative size of samples. Grey boxes indicate ORs and CIs for the overall cohort of MI patients, black boxes for patients represented with a positive family history.

Statistical analyses revealed odds ratios in the range of 1.892 to 2.769 for MI patients with a positive family history demonstrating a much stronger association than for the overall cohort of MI patients and therefore a considerable increased risk for MI patients with a family burden of the disease (Figure 1).

These findings could be confirmed comparing MI patients with a positive family history with patients without a positive family history for all SNP variants with the exception of the rs1333040 SNP (Table 4).

**Figure 2:**
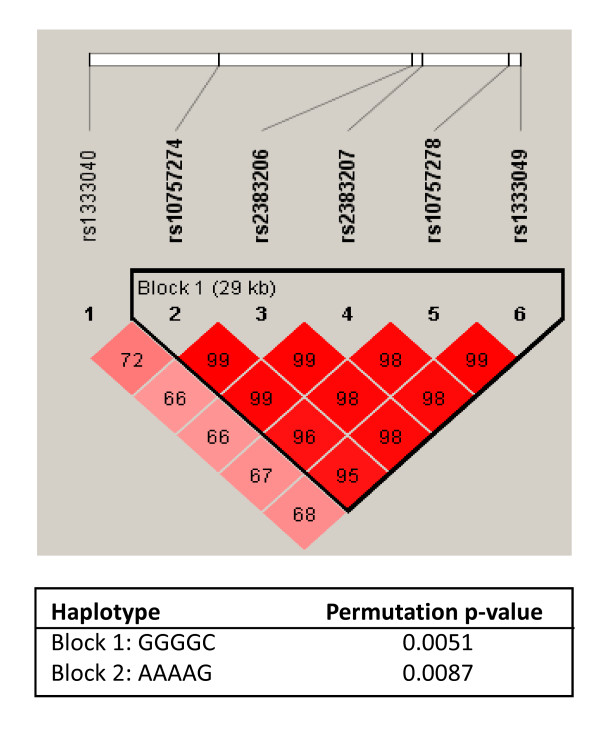
**Haplotype analysis for the investigated SNPs**. As can be seen, all SNPs with the exception of the rs1333040 show a positive association of the phenotype MI in the presents of a positive family history of the disease. The haplotypes GGGGC and AAAAG revealed the strongest association of MI with a positive family history.

These results could also be confirmed in an additional haplotype analysis (Figure 2). For the haplotypes GGGGC and AAAAG spanning the rs10757274, rs2383206, rs2383207, rs10757278, and rs1333049 SNPs a significant association of a positive vs. a negative family history could be shown (p = 0.0051 and p = 0.0087 respectively). Adjustment for other cardiovascular risk factors (smoking, body mass index, hypertension, diabetes mellitus and hyperlipidemia) revealed no higher frequencies of the risk variants within the 9p21.3 region in MI patients examined by means of subgroup analyses (data not shown). These findings emphasize this genetic region as a genetic risk factor for MI independent of other risk factors.

## Discussion

In recent years, genome wide association studies focused on genomic factors involved in development of CAD and MI. In these studies and subsequent replication studies the 9p21.3 locus was found to be the most common locus associated with CAD and MI. Moreover, in a subsequent study, a strong association of the SNP rs1333049 representing the 9p21.3 risk variant with the extent of CAD could be shown[18].

In the present study, we investigated the association of six previously reported sequence variants representing the 9p21.3 locus in a large scale study population of male patients who had suffered from an acute MI. In this well defined study cohort, the exclusive inclusion of patients aged ≤ 65 years increased the probability that genetic factors are involved to a significant degree in the onset of MI. Moreover, only men were enrolled which excludes gender specific differences having an influence on the development of CHD. It is well known that women develop CHD about 10 years later than men, probably due to the protective effects of female sex hormones but also due to the different frequency of classical risk factors such as diabetes, hypertension and smoking habits[19,20].

Genotyping of the study population showed similar or even higher high-risk allele frequencies compared to those in previous studies which reported the association of 9p21.3 sequence variants with CHD and MI[3,4,11]. Comparison was carried out using previously cited control subjects from central or northern Europe and without a known history of CAD. Moreover, in the German PopGen study only men were used as control subjects[11]. Thus, the selected controls were comparable to the present MI cohort and therefore justifiably applied in the presented association analyses. Comparison of the data with control subjects revealed a statistical significant difference in the genotype distribution and allelic frequencies of all analyzed polymorphisms within the 9p21.3 region. The observed higher odds ratios are probably due to the much more homogeneity of the study cohort investigated in the present study. No significant difference was observed in the genotype distribution of our MI cases and MI patients from previous studies. Hence, we confirmed the association of this locus with MI in the present study cohort. This emphasises once more the relevance of this genetic factor in the pathogenesis of CAD and MI.

Having replicated the association of all investigated SNPs with MI, patients having definitively presented with positive family history of MI were selected and compared to the corresponding control cohorts or a subpopulation of patients with a definitively negative family history. For the accuracy of this analysis, patients who provided no information on a family history of MI were excluded. This analysis revealed a significantly stronger association in patients having presented with a positive family history of MI than in the overall cohort of MI patients with calculated odds ratios within the range of 1.892 to 2.769.

Classification according to a positive family history provides an improved risk prediction for the patients. In fact, the increased risk of carriers of the homozygous risk alleles and a positive family history is comparable to the increased risk of hypertensive patients (OR 1.91), patients with diabetes mellitus (OR 2.37) or current smokers (OR 2.87) calculated in a large case-control study of acute MI in 52 countries[21].

## Conclusions

To our knowledge the present study is the first one to analyse the association of all six 9p21.3 SNPs with acute MI in relation to a positive family history. Indeed, few other studies reported the association with CAD after classification of patients according to their family history. However, our study differs from the others in the origin of the patients, in the strict inclusion criteria and in the larger number of patients. Moreover, the quantity of analyzed sequence variants in the present study was higher.

Our findings made evident the correlation between high-risk variants on the 9p21.3 locus and male patients with acute MI and a positive family history. Over the past few years, risk evaluation has exclusively been performed on the prevalence of classical risk factors such as age and gender, LDL-cholesterol, hypertension, diabetes mellitus, smoking habits, diet, a family history and physical activity[22,23]. The findings of the present study may help to improve risk assessment and early prevention particularly for patients with a positive family history of CAD when 9p21.3 sequence variants are taken into account.

## Limitations of the study

One limitation of the study is the lack of an own control group. Using previous reported data about the allelic distribution of SNP's will be of a limited bias due to the fact, that thousands of chromosomes where analysed in previous reported studies. Another limitation is that not a consistent control group could be used for comparison.

## Competing interests

The authors declare that they have no competing interests.

## Authors' contributions

TS, MS, AH and GH have made substantial contributions to conception and design as well as analysis and interpretation of the data. KS and PB have made the genetic analyses. HRO, WS, JT, WM, F-JH, ChS, ThD, HG, HH, WD acquired patients and clinical data, TS and KS wrote the manuscript. All authors have read and approved the final version of the manuscript.

## Pre-publication history

The pre-publication history for this paper can be accessed here:

http://www.biomedcentral.com/1471-2261/11/9/prepub
